# *Asterodiscus* and *Stigmatodiscus*, two new apothecial dothideomycete genera and the new order *Stigmatodiscales*

**DOI:** 10.1007/s13225-016-0356-y

**Published:** 2016-02-02

**Authors:** Hermann Voglmayr, Alain Gardiennet, Walter M. Jaklitsch

**Affiliations:** 1 Division of Systematic and Evolutionary Botany, Department of Botany and Biodiversity Research, University of Vienna, Rennweg 14, A-1030 Vienna, Austria; 2 14 rue Roulette, F-21260 Véronnes, France; 3 Institute of Forest Entomology, Forest Pathology and Forest Protection, Department of Forest and Soil Sciences, BOKU-University of Natural Resources and Life Sciences, Hasenauerstraße 38, A-1190 Vienna, Austria

**Keywords:** *Ascomycota*, *Dothideomycetes*, New species, New genus, New family, New order, Phylogenetic analysis, Taxonomy

## Abstract

During a survey on corticolous *Dothideomycetes*, several collections with ascospores matching the genera *Asteromassaria* and *Stigmatomassaria* (*Pleomassariaceae*, *Pleosporales*) were revealed from dead corticated twigs of *Acer*, *Carpinus* and *Tamarix*. Closer morphological examination showed that their ascomata were apothecial, with a hamathecium consisting of septate, branched paraphyses, which are apically swollen at maturity. Several collections were cultured and sequenced, and a Blast search of their nuc 28S rDNA sequences revealed dothideomycetous affiliation, but without a close match to a specific family or order. Phylogenetic analyses of a multigene matrix containing a representative selection of *Dothideomycetes* from four genes (nuc 18S rDNA, nuc 28S rDNA, *rpb*2 and *tef*1) revealed placement within *Dothideomycetes* but without a supported familial or ordinal affiliation. Based on the phylogenetic analyses and morphological investigations, the new genera *Asterodiscus* and *Stigmatodiscus*, with the two new species *A. tamaricis* and *S. enigmaticus*, are described and illustrated, and placed in the new family *Stigmatodiscaceae* and new order *Stigmatodiscales*.

## Introduction

During a survey on corticolous ascomycetes in Istria, Croatia in September 2010, we collected a dothideomycetous species from dead corticated twigs of *Carpinus orientalis* which showed ascospores with a striking resemblance to *Stigmatomassaria pupula* (*Pleomassariaceae*, *Pleosporales*; see Barr 1982, under *Splanchnonema pupula*). They were first considered to represent poorly developed, aberrant ascomata of *Stigmatomassaria*, but detailed morphological examination showed that the ascomata were clearly apothecial, with ascomata opening at maturity and having septate paraphyses with apical swellings at their free ends. Sequence data obtained from cultures of ascospores left no doubt that they do not belong to *Stigmatomassaria* or even *Pleosporales*. NCBI Blast searches confirmed dothideomycetous affinities, but without indication of a closer relative. Subsequently, we collected and cultured the fungus several times from *Acer monspessulanum*, *A. campestre* and *A. sempervirens* from various European countries.

In summer 2013, we found another dothideomycetous species on corticated dead twigs of *Tamarix* spp. with ascospores matching the genus *Asteromassaria* (*Pleomassariaceae*, *Pleosporales*; see Barr 1982), but again with apothecial ascomata having apically swollen paraphyses. Sequence data obtained from pure cultures revealed a close phylogenetic relationship to the *Stigmatomassaria*-like apothecial dothideomycete.

These fungi could not be identified upon consultation of the taxonomic literature, and no genera or families offered themselves for an appropriate taxonomic placement. Therefore, they are described in the new genera *Asterodiscus* and *Stigmatodiscus*, which are placed in a new family and order within *Dothideomycetes*, according to the results of multigene phylogenetic analyses and detailed morphological investigations.

## Materials and methods

### Morphological observations

Hand sections of ascomata were made using a razor blade and mounted in water on a microscope slide, gently torn apart with a preparation needle when necessary and covered with a cover slip. Slides were examined and photographed using a Zeiss Axio Imager.A1 (Zeiss, Jena, Germany) microscope equipped with a Zeiss AxioCam ICc3 or Axiocam 506 colour digital camera. Measurements are reported as maxima and minima in parentheses and the mean plus and minus the standard deviation given in parentheses. For falcate conidia, the shortest distance between both ends (i.e. diameter) was measured. The specimens were deposited in the fungarium of the University of Vienna (WU).

### Pure culture isolation

Mature ascomata on corticated twigs were horizontally cut using a sterile razor blade, the apothecia separated from the surrounding host tissue, transferred in a sterile drop of water on a microscope slide, torn apart with a forceps to release the ascospores from asci and pipetted on a 2 % malt extract agar (MEA) plate supplemented with 200 mg/L penicillin G and streptomycin sulphate (Sigma-Aldrich, St. Louis, MO). Germinated ascospores were then transferred to 2 % MEA plates, which were sealed with laboratory film and incubated at room temperature. Cultures were deposited at CBS-KNAW Fungal Biodiversity Centre, Utrecht, The Netherlands (CBS).

### DNA extraction, PCR and sequencing

Growth of liquid cultures and extraction of genomic DNA was done according to Voglmayr and Jaklitsch (2011), either using the modified CTAB extraction protocol of Riethmüller et al. (2002) or the DNeasy Plant Mini Kit (QIAgen GmbH, Hilden, Germany). The following loci were used for identification and phylogenetic analyses: The complete ITS region and D1 and D2 domains of 28S nuc rDNA region (ITS-28S) were amplified using the primers V9G (de Hoog and de and Gerrits van den Ende 1998) and LR5 (Vilgalys and Hester 1990). The 18S nuc rDNA region was amplified with primers SL1 (Landvik et al. 1997) and NS24mod (Voglmayr and Jaklitsch 2011). A ca 1.1 kb fragment of the RNA polymerase II subunit 2 (*rpb*2) gene was amplified using the primer pair fRPB2-5f and fRPB2-7cr (Liu et al. 1999). A ca 1.3 kb fragment of translation elongation factor 1-α (*tef*1) gene was amplified with the primers EF1728F (Carbone and Kohn 1999) and TEF1LLErev (Jaklitsch et al. 2005). The latter fragment includes the fourth and the fifth intron and a significant portion of the last large exon. A ca. 0.8 kb fragment of the β-tubulin (*tub*2) gene was amplified with primers T1HV (5′ CANMATGCGYGAGATYGTAYGT 3′) and BtHV2r (5′ CATCATRCGRTCNGGGAACTC 3′), which were newly developed for *Dothideomycetes*. T1HV is a modified version of primer T1 (O’Donnell and Cigelnik 1997), whereas BtHV2r is upstream from T222 (O’Donnell and Cigelnik 1997); the primers were developed from genome sequences of representative *Dothideomycetes* downloaded from the Joint Genome Institute (JGI, http://jgi.doe.gov/). PCR products were purified using an enzymatic PCR cleanup (Werle et al. 1994) as described in Voglmayr and Jaklitsch (2008). DNA was cycle-sequenced using the ABI PRISM Big Dye Terminator Cycle Sequencing Ready Reaction Kit v. 3.1 (Applied Biosystems, Warrington) and the PCR primers; in addition, the following primers were used: ITS-28S region: ITS4 (White et al. 1990), LR3 (Vilgalys and Hester 1990); SSU: NS1088 (Kauff and Lutzoni 2002). Sequencing was performed on an automated DNA sequencer (ABI 3730xl Genetic Analyzer, Applied Biosystems).

### Phylogenetic analyses

To reveal the phylogenetic position of the two new genera, a matrix of aligned nucleotide sequences from four different phylogenetic markers (18S, 28S, *rpb*2 and *tef*1), and 219 representatives of *Dothideomycetes* and *Eurotiomycetes*, including two species from *Lecanoromycetes* as outgroup, was produced. The matrix of Boehm et al. (2015) was downloaded from TreeBASE (www.treebase.org; submission No. S 16,151) and served as the matrix basis after removal of a few sequences. Sequences of six additional taxa were downloaded from the GenBank nucleotide database. These, together with the newly generated sequences, were manually aligned to the matrix of Boehm et al. (2015), or realigned with the server version of MAFFT (www.ebi.ac.uk/Tools/mafft). The resulting alignments were subsequently checked and refined using BioEdit version v. 7.0.4.1 (Hall 1999). For alignment of *rpb*2, first the alignment of Boehm et al. (2015) was translated into a protein matrix which was then re-aligned with the new protein sequences using MAFFT, and the respective new DNA sequences were then manually aligned to the original *rpb*2 nucleotide matrix using the protein alignment as reference. Prior to phylogenetic analyses, the approach of Wiens (1998) was applied to test for significant levels of localized incongruence among the four genes, using the level of bootstrap support (Sung et al. 2007). For this, the 80 % maximum likelihood (ML) bootstrap consensus trees for each individual partition were compared using the same parameters as for the combined analysis given below, but using 100 fast bootstrap replicates. No significant topological conflicts were observed between the bootstrap trees of 18S, 28S, *rpb*2 and *tef*1, indicating the absence of significant incongruence and combinability of the matrices (Wiens 1998). The resulting combined sequence matrix contained 5721 nucleotide positions from four genes (1653 from 18S, 1230 from 28S, 1875 from *rpb*2, 963 from *tef*1). GenBank accession numbers of newly generated sequences are given in Table 1.

**Table 1:** Isolates and GenBank accession numbers generated during the present study

Taxon	Origin	Host	Voucher	Isolate	GenBank accession numbers				
					SSU	ITS-LSU	*tef*1	*rpb*2	*tub*2
*Asterodiscus tamaricis*	Austria, Wien, Landstraße	*Tamarix tetrandra*	WU 35906	L114 = CBS 136919	KU234128	KU234101	KU234133	KU234116	KU234135
*A. tamaricis*	Croatia, Istria, Rovinj	*Tamarix* sp.	WU 35921	L161		KU234103			
*A. tamaricis*	France, Bourgogne, Côte-d’Or, Lux	*Tamarix gallica*	WU 35908	L113 = CBS 136918		KU234100	KU234132	KU234115	KU234134
*A. tamaricis*	Italy, Lazio, Viterbo, Vetralla	*Tamarix* sp.	WU 35910	L124		KU234102		KU234117	KU234136
*Stigmatodiscus enigmaticus*	Austria, Wien, Donaustadt, Lobau	*Acer campestre*	WU 35913	L84		KU234114		KU234127	KU234146
*S. enigmaticus*	Austria, Wien, Landstraße	*Acer monspessulanum*	WU 35914	L69 = CBS 132036	KU234130	KU234108		KU234121	KU234140
*S. enigmaticus*	Croatia, Istria, Krnica	*Carpinus orientalis*	WU 35915	L68		KU234107		KU234120	KU234139
*S. enigmaticus*	Croatia, Istria, St. Golaš	*Carpinus orientalis*	WU 35916	L71 = CBS 131997		KU234109		KU234122	KU234141
*S. enigmaticus*	Czech Republic, Morava, Lednice	*Acer monspessulanum*	WU 35917	L64	KU234129	KU234106		KU234119	KU234138
*S. enigmaticus*	France, Alpes-de-Haute-Provence, Ganagobie	*Acer monspessulanum*	WU 35918	L76 = CBS 132037		KU234111		KU234124	KU234143
*S. enigmaticus*	France, Var, Gorges du Verdon	*Acer monspessulanum*	WU 35919	L75		KU234110		KU234123	KU234142
*S. enigmaticus*	Greece, Crete, Chania, Omalos	*Acer sempervirens*	WU 35911	L82		KU234112		KU234125	KU234144
*S. enigmaticus*	Greece, Crete, Chania, Omalos	*Acer sempervirens*	WU 35912	L83	KU234131	KU234113		KU234126	KU234145
*S. enigmaticus*	Italy, Lazio, Viterbo, Norchia	*Acer campestre*	WU 35920	L122		KU234104		KU234118	KU234137
*S. enigmaticus*	Slovenia, Sežana, Skocjan	*Acer monspessulanum*	WU 35922	L162		KU234105			

For detailed phylogenetic analyses within the new family, the ITS-28S, *rpb*2 and *tub*2 sequences of all accessions sequenced were aligned and combined in a single three-loci matrix; for GenBank accession numbers see Table 1. This three-loci matrix contained 3657 nucleotide positions (1654 from ITS-28S, 1168 from *rpb*2 and 835 from *tub*2).

For maximum likelihood (ML) analyses and Bayesian inference (BI) of the multi-gene analysis of *Dothideomycetes*, unique model parameters were applied for each marker and codon (where applicable), with the dataset divided in eight partitions according to Schoch et al. (2009a). The general time reversible model, assuming a proportion of invariant sites and gamma-distributed substitution rates (GTR + I + G), was selected as best model for all four genes (LSU, SSU, *rpb*2 and *tef*1) by Modeltest 3.6 (Posada and Crandall 1998) under the Akaike Information Criterion (AIC) and was implemented in the subsequent ML and BI analyses. The matrix of *Stigmatodiscaceae* was divided in three partitions according to the three loci.

For ML analyses, fast tree searches were done with RAxML (Stamatakis 2006) as implemented in raxmlGUI 1.3 (Silvestro and Michalak 2012), using the GTRGAMMAI substitution model. 500 fast bootstrap replicates were computed using the GTRCATI substitution model.

Bayesian analyses were performed on the large matrix of *Dothideomycetes* with the computer program MrBayes (version 3.2.2; Huelsenbeck and Ronquist 2001). Three parallel runs of four incrementally heated, simultaneous Markov chains were performed over 10 million generations, of which every 1000th tree was sampled in each run, using the GTR + I + G model for all eight partitions. The first 4000 of the 10,000 trees sampled were discarded, and a 90 % majority rule consensus of the remaining trees was computed to obtain posterior probabilities (PP).

Maximum parsimony (MP) analyses were performed with PAUP v. 4.0a142 (Swofford 2002) only for the small three-loci matrix of *Stigmatodiscaceae*, using 1000 replicates of heuristic search with random addition of sequences and subsequent TBR branch swapping (MULTREES option in effect, steepest descent option not in effect). All molecular characters were unordered and given equal weight; analyses were performed with gaps treated as missing data; the COLLAPSE command was set to NO. Bootstrap analysis with 1000 replicates was performed in the same way, but using 10 rounds of random sequence addition and subsequent TBR branch swapping during each bootstrap replicate.

## Results

### Molecular phylogeny

**Fig. 1:**
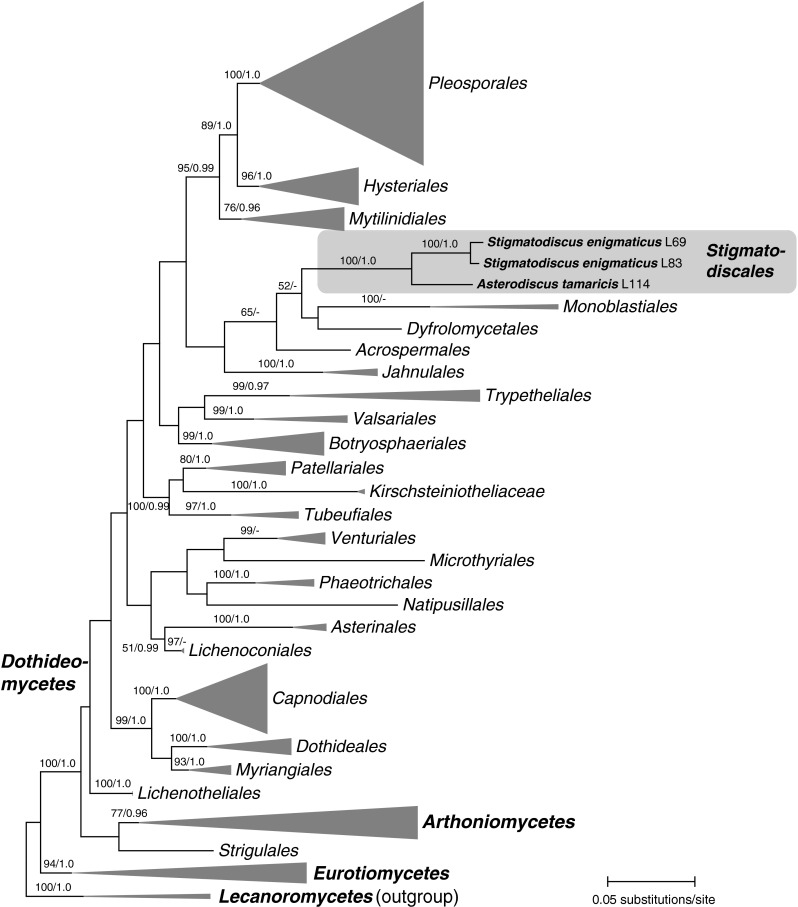
Simplified phylogram showing the best RAxML maximum likelihood tree (lnL = −137,324.200031) obtained from the combined multigene (LSU, SSU, *rpb*2, *tef*1) matrix of 219 taxa including major orders in *Dothideomycetes*, *Arthoniomycetes* and *Eurotiomycetes*, with two members of *Lecanoromycetes* (*Cladonia caroliniana*, *Flavoparmelia caperata*) selected as outgroup according to Boehm et al. (2015). Ordinal and familial classification follows Hyde et al. (2013) and Boehm et al. (2015). *Stigmatodiscus* and *Asterodiscus* form a highly supported clade representing a distinct order within *Dothideomycetes*. Except for *Stigmatodiscales*, all lineages were collapsed to ordinal or familial level. ML bootstrap support above 50 % and Bayesian posterior probabilities above 0.9 are given above or below the branches

No *tef*1 sequences could be obtained for *Stigmatodiscus*, despite various attempts using different primer sets, polymerases or PCR protocols; therefore, this gene could only be included for *Asterodiscus* in the multi-gene analyses of *Dothideomycetes*. Of the 5721 nucleotide positions included in the *Dothideomycetes* matrix, 2595 were parsimony informative (488 from 18S, 507 from 28S, 1198 from *rpb*2, 402 from *tef*1). The best ML tree revealed by the RAxML analysis of the matrix including representatives of all major orders and families of *Dothideomycetes*, *Arthoniales*, *Trypetheliales* and *Eurotiomycetes* is shown as phylogram in Fig. 1, with the orders/families collapsed to enable a better overview. As observed in previous studies on *Dothideomycetes* (e.g. Schoch et al. 2009a, 2009b, Hyde et al. 2013; Wijayawardene et al. 2014; Boehm et al. 2015), many of the deeper nodes receive insignificant or low internal support, whereas many of the orders and families are highly supported. *Asterodiscus* and *Stigmatodiscus* form a distinct highly supported clade within *Dothideomycetes* and are contained in a clade with *Monoblastiales*, *Dyfrolomycetales* and *Acrospermales*, but only with low ML bootstrap support (65 %). Sister group relationship of the *Asterodiscus*-*Stigmatodiscus* clade to the *Monoblastiales*-*Dyfrolomycetales* clade receives low ML bootstrap support as well (52 %), which means that a statistically well-supported close relationship to other orders within *Dothideomycetes* remains obscure at this time.

**Fig. 2:**
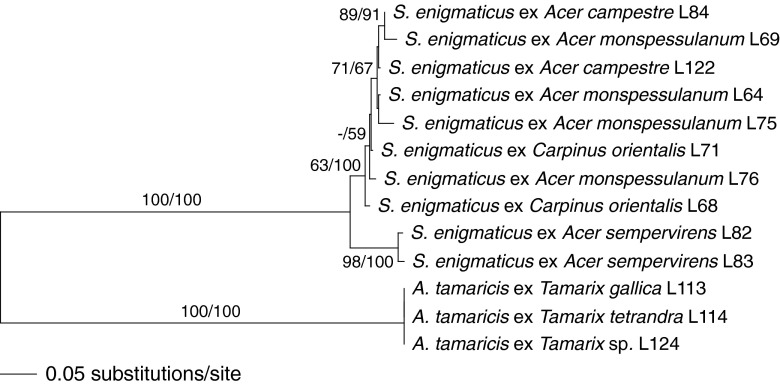
Phylogram the of best RAxML maximum likelihood tree (lnL = −7783.3102) obtained from the combined multigene matrix of ITS-LSU, *rpb*2 and *tub*2 from *Asterodiscus* and *Stigmatodiscus*. ML and MP bootstrap values above 50 % are given above or below the branches

Of the 5721 nucleotide positions included in the three-loci matrix of *Stigmatodiscaceae*, 476 were parsimony informative (148 from 28S-ITS, 208 from *rpb*2, 120 from *tub*2). The MP analysis revealed 12 MP trees of score 580; in the strict consensus tree, all nodes lacking MP bootstrap support collapsed to a polytomy (data not shown). Figure 2 shows the best ML tree of the detailed phylogenetic analysis of *Stigmatodiscaceae* revealed by RAxML, which is fully compatible with the MP strict consensus tree. Within *Stigmatodiscus enigmaticus*, some sequence variability was observed between the various accessions included in the analyses. The accessions from Crete on *Acer sempervirens* were set apart as sister group to the other accessions with moderate (ML) to high (MP) support. There is no further grouping according to hosts or geographic origins, and the lack of morphological differentiation indicates that only a single *Stigmatodiscus* species is involved. The sequences of all three *Asterodiscus* accessions from Austria, France and Italy were highly similar to identical.

### Taxonomy

***Stigmatodiscales*** Voglmayr & Jaklitsch, ***ord. nov.***

MycoBank MB 815325.

Type family: *Stigmatodiscaceae* Voglmayr & Jaklitsch.

*Ascomata* apothecioid, embedded in cortex of dead twigs, without distinct margin nor excipulum. *Hamathecium* paraphysate; paraphyses septate, unbranched or rarely branched and anastomosing above, with free apical ends, covered by an epithecium. *Asci* bitunicate, fissitunicate, I-, without a ring. *Ascospores* large, slightly to strongly asymmetric, hyaline to brown, multiseptate with one euseptum and several distosepta, wall surrounded by gel coating. *Conidiomata* in nature immersed, peridermal, pycnidial.

***Stigmatodiscaceae*** Voglmayr & Jaklitsch, ***fam. nov.***

MycoBank MB 815326.

Type genus: *Stigmatodiscus* Voglmayr & Jaklitsch.

*Ascomata* apothecioid, embedded in cortex of dead twigs, dark brown to black, without distinct margin. *Hamathecium* paraphysate; paraphyses septate, unbranched or rarely branched and anastomosing above, with swollen free apical ends, embedded in a rubber-like gel matrix and covered by an epithecium. *Hymenial gel* I-. *Asci* sequentially produced over a long time, bitunicate, fissitunicate, I-, broadly fusoid to saccate, with thin ecto- and thick endotunica, apically with wide ocular chamber, without a ring. *Ascospores* slightly to strongly asymmetric, distal part larger than proximal part, hyaline to brown, 1-euseptate in early stages, developing 2 additional distosepta, wall surrounded by gel coating. *Conidiomata* in nature immersed, peridermal, pycnidial.

***Asterodiscus*** Voglmayr, Gardiennet & Jaklitsch, ***gen. nov.***

MycoBank MB 815329, Facesoffungi number: FoF 01656.

*Etymology*: Referring to the striking similarity of its ascospores to those of *Asteromassaria*.

Type species: *Asterodiscus tamaricis* Voglmayr, Gardiennet & Jaklitsch.

*Ascomata* embedded in cortex of dead twigs, initially covered by bark, lifting the bark as a black bump, globose-depressed to broadly pyriform, apically covered by black tissue of *textura intricata*, at maturity apothecioid, breaking open in the centre with irregular radial cracks; finally a depressed disc becoming fully exposed with age, sometimes confluent with neighbouring discs, dark brown to black, without distinct margin. *Hamathecium* of hyaline, septate paraphyses, often branched and anastomosing above, with distinctly swollen free apical ends, embedded in a rubber-like gel matrix and covered by an amorphous matrix forming an epithecium. *Hymenial gel* I-. *Asci* forming sequentially and slowly, bitunicate, fissitunicate, I-, variable in shape from broadly fusoid to saccate, with thin ecto- and thick endotunica; apex with a wide ocular chamber, without ring. *Ascospores* large, 30–50 μm long, asymmetric, distal part slightly larger than the proximal part, ends rounded to subacute, hyaline to light brown with age, first 1-euseptate, developing 2 additional distosepta and becoming 3-septate, distinctly constricted at the septa, secondary septa with large pores; wall thick, smooth to slightly verruculose, surrounded by gel coating widely expanding in water after spore discharge; lumina ellipsoid-rectangular in mid cells, semiglobose in end cells. *Asexual morph* in pure culture acervular, superficial, aggregated, conidiogenous cells phialidic, conidia thread-like, sinuously curved to allantoid.

***Asterodiscus tamaricis*** Voglmayr, Gardiennet & Jaklitsch, ***sp. nov.***

**Fig. 3:**
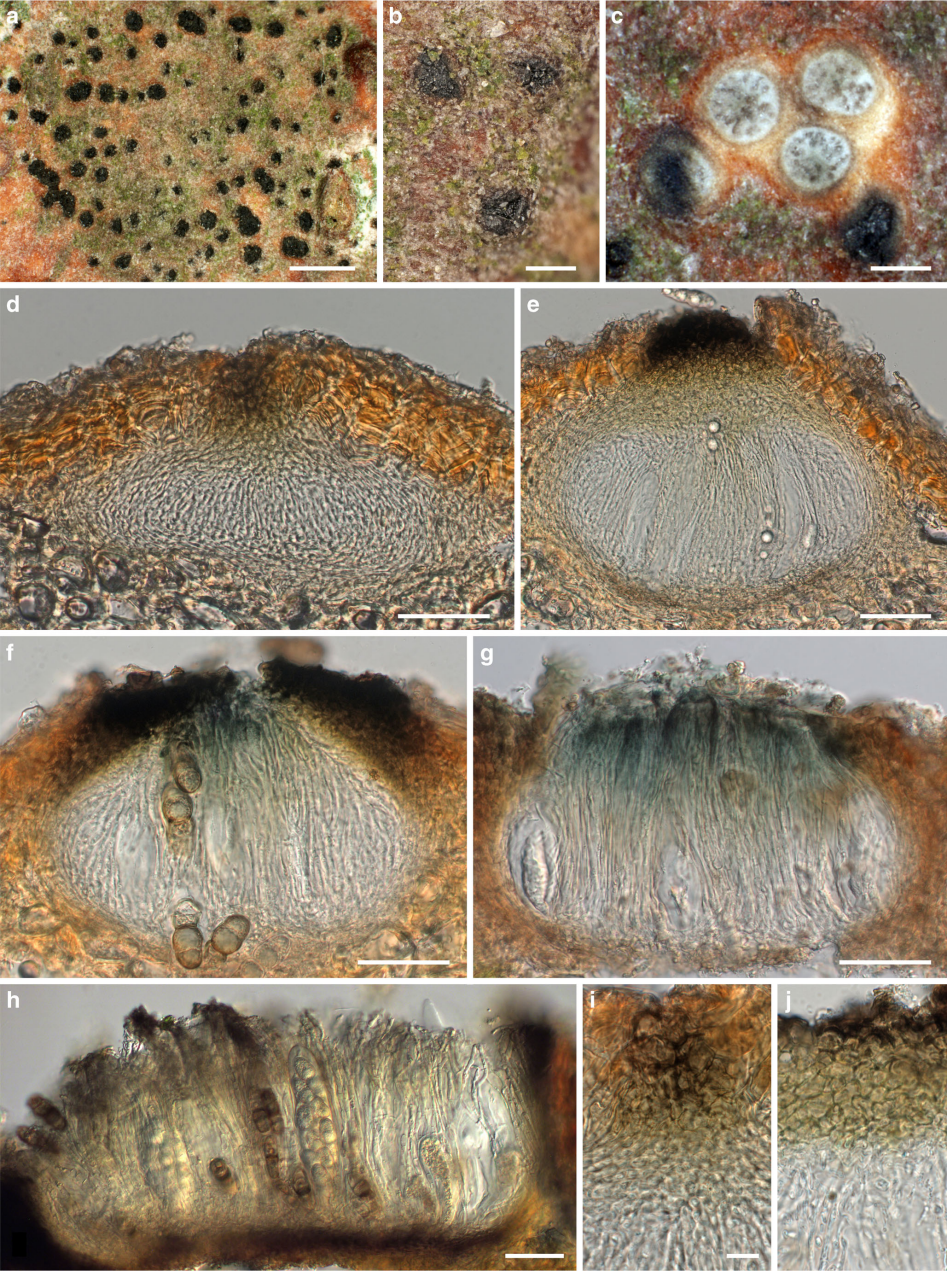
*Asterodiscus tamaricis*, sexual morph. **a, b** Ascomata erumpent from bark in face view. **c** Ascomata in transverse section, showing the hymenium with asci. **d–h** Vertical sections of ascomata embedded in bark; **d, e** closed young ascomata showing the hamathecial threads connected with apical dark brown tissue of *textura intricata*; **f–h** apothecioid ascomata with paraphyses and asci embedded in a gel matrix stained bluish (**f, g**) to olivaceous (**h**) below the epithecium. **i, j** Olivaceous to dark brown epithecium of young ascomata forming a *textura intricata* and young paraphyses below; (**i**) detail from **d**. All in water. *Sources*: *a*, *c*, *h*. WU 35908; *b*. WU 35907; *d*–*g*, *i*, *j*. WU 35924. *Scale bars*: *a* = 1 mm. *b* = 250 μm. *c* = 100 μm. *d*–*h* = 50 μm. *i*, *j* = 10 μm

**Fig. 4:**
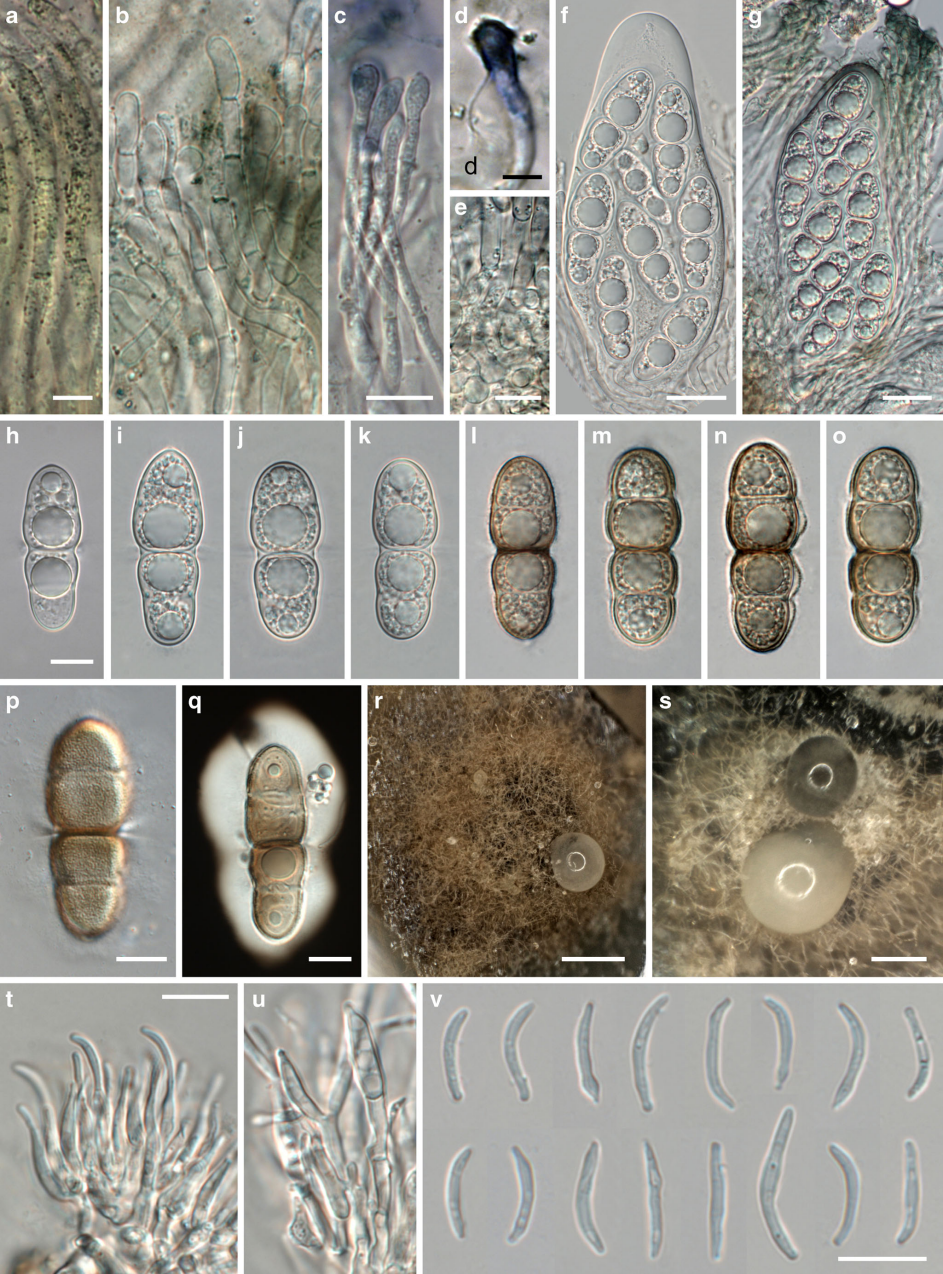
*Asterodiscus tamaricis*, sexual (**a–q**) and asexual (**r–v**) morph. **a–d** Apically inflated septate paraphyses, covered by an olivaceous (**a, b**) to deep blue (**c, d**) amorphous incrustation. **e** Subhymenium and bases of paraphyses. **f, g** Asci, in **g** embedded in gel matrix with paraphyses. **h–k** Hyaline ascospores. **l–q** Brown ascospores after ejection; in **p** showing verruculose wall ornamentation, in **q** showing gel sheath. **r, s** Acervular conidiomata on MEA showing conidial drops. **t, u** Phialides on branched conidiophores. **v** Conidia. All in water; **q** in black ink. *Sources*: *a*, *c*, *d.* WU 35908, *b*, *e*, *g*, *m*–*q*. WU 35924; *f*, *i*–*l*. WU 35907; *h*. WU 35906 (holotype); *r*–*v*. WU 35921. *Scale bars*: *a*, *b*, *d* = 5 μm. *c*, *e*, *h*–*q*, *r*–*v* = 10 μm. *f*, *g* = 20 μm. *r* = 400 μm. *s* = 200 μm

MycoBank MB 815330, Facesoffungi number: FoF 01657, Figs. 3, 4.

*Etymology*: Referring to its occurrence on *Tamarix* spp.

*Ascomata* apothecioid, embedded in cortex of dead twigs, initially covered by bark, emerging as a black bump 130–200 μm diam when young, later upper layer breaking open in the centre with irregular radial cracks, finally a circular to elongated flat to depressed, 200–600 μm wide disc becoming fully exposed, sometimes confluent with neighbouring discs, dark brown to black, without distinct margin. *Subhymenium* thin, ca 15–20 μm thick, brown, of tightly packed subhyaline to light brown hyphae 3–8 μm diam. *Paraphyses* 80–150 μm long, 2–3.5 μm wide, hyaline, septate, cells sometimes becoming inflated up to 7 μm with age, with free apical ends swollen, club-like, up to 9 μm wide, apical swellings with age incrusted by a deep blue, finely granular stain, covered by an olivaceous, emerald to deep blue amorphous incrustation. *Hymenial gel* in lower part hyaline, in upper part dark olivaceous, emerald to deep blue. *Asci* (73–)96–144(−163) × (35–)42–59(−72) μm (*n* = 26), variable in shape from broadly fusoid to saccate, thick-walled, containing 8 irregularly bi- to triseriate ascospores. *Ascospores* (33.4–)39.8–45(−48.8) × (12.8–)14.3–16.5(−17.7) μm, l/w = (2.4–)2.6–2.9(−3.2) (*n* = 90), asymmetric, first 1-septate, developing 2 additional distosepta and becoming 3-septate with age, strongly constricted at the primary septum, weakly at secondary septa, secondary septa with large pores, hyaline to light brown with age, becoming dark brown after ejection, ends mostly rounded, distal ends sometimes subacute, surrounded by a thick gelatinous sheath, wall smooth to finely verruculose, the contents granular, with a large, strongly refractive guttule per cell.

*Conidiomata* on the natural substrate not observed, on 2 % MEA acervular, superficial, aggregated, ca 200–450 μm wide, conidiophores densely aggregated, branched. *Conidiogenous cells* phialidic, (7.2–)10.2–15.3(−20.4) × (1.8–)2.1–3.2(−3.8) μm (*n* = 30), lageniform. *Conidia* irregularly sinuously curved to falcate, (9.4–)10.6–13.6(−17.4) × (1.0–)1.2–1.6(−2.1) μm (*n* = 70), hyaline, smooth. *Cultures* very slow-growing, with uneven margins, colony on 2 % MEA reaching 32 mm diam after one month at room temperature, first whitish, turning dark brown, with whitish aerial hyphae in the centre, reverse first hazelnut brown, then black; on PDA colony light brown, cottony, with the centre covered by whitish mycelium, reverse hazelnut brown with dark brown centre.

*Holotype*: AUSTRIA, Wien, Landstraße, Botanical Garden of the University (HBV), on *Tamarix tetrandra*, 1 Aug 2013, H. Voglmayr (WU 35906, ex-type culture CBS 136919 = L114).

*Additional specimens examined*: AUSTRIA, Wien, Landstraße, Botanical Garden of the University (HBV), on *Tamarix tetrandra*, 7 May 2015, H. Voglmayr (WU 35924). CROATIA, Istria, Rovinj, Kamp Amarin, on *Tamarix* sp., 15 May 2015, H. Voglmayr, M. & I. Greilhuber (WU 35921, culture L161). FRANCE, Bourgogne, Côte-d’Or (21), Is-sur-Tille, route de Dijon, on *Tamarix gallica*, 25 Apr 2015, A. Gardiennet AG15034 (WU 35907). Lux, on *Tamarix gallica*, 21 Jul 2013, R. Rousseaux & A. Gardiennet AG13137 (WU 35908, culture L113 = CBS 136918). Véronnes, on *Tamarix gallica*, 16 Apr 2015, A. Gardiennet AG15029 (WU 35909). ITALY, Lazio, Viterbo, Vetralla, on *Tamarix* sp., 14 Oct 2013, W. Jaklitsch, H. Voglmayr & W. Gams (WU 35910, culture L124).

***Stigmatodiscus*** Voglmayr & Jaklitsch, ***gen. nov.***

MycoBank MB 815327, Facesoffungi number: FoF 01654.

*Etymology*: Referring to the striking similarity of its ascospores to those of *Stigmatomassaria*.

Type species: *Stigmatodiscus enigmaticus* Voglmayr & Jaklitsch.

*Ascomata* apothecioid, embedded in cortex of dead twigs, initially covered by bark, later becoming exposed through irregular cracks, eventually becoming fully exposed with age, dark brown to black, without distinct margin. *Hamathecium* of hyaline, septate paraphyses, unbranched or rarely branched and anastomosing above, with distinctly swollen free apical ends, embedded in a rubber-like gel matrix and covered by an amorphous matrix forming an epithecium. *Hymenial gel* I-. *Asci* sequentially produced over a long time, bitunicate, fissitunicate, I-, variable in shape from broadly fusoid to saccate, with thin ecto- and thick endotunica, apically with wide ocular chamber, without ring. *Ascospores* large, 45–80 μm long, asymmetric, distal part distinctly larger than the proximal part, proximal end rounded, distal end rounded to subacute, brown, 1-euseptate in early stages, developing 2 additional distosepta and becoming 3- septate, distinctly constricted at the septa, secondary septa with large pores; wall thick, punctate to verruculose, surrounded by gel coating widely expanding in water after spore discharge; lumina ellipsoid in mid cells, ellipsoid to conoid in end cells. *Conidiomata* on natural substrates immersed, peridermal, pycnidial, thin-walled, on 2 % MEA superficial, acervular, condiogenous cells phialidic, conidia falcate.

***Stigmatodiscus enigmaticus*** Voglmayr & Jaklitsch, ***sp. nov.***

**Fig. 5:**
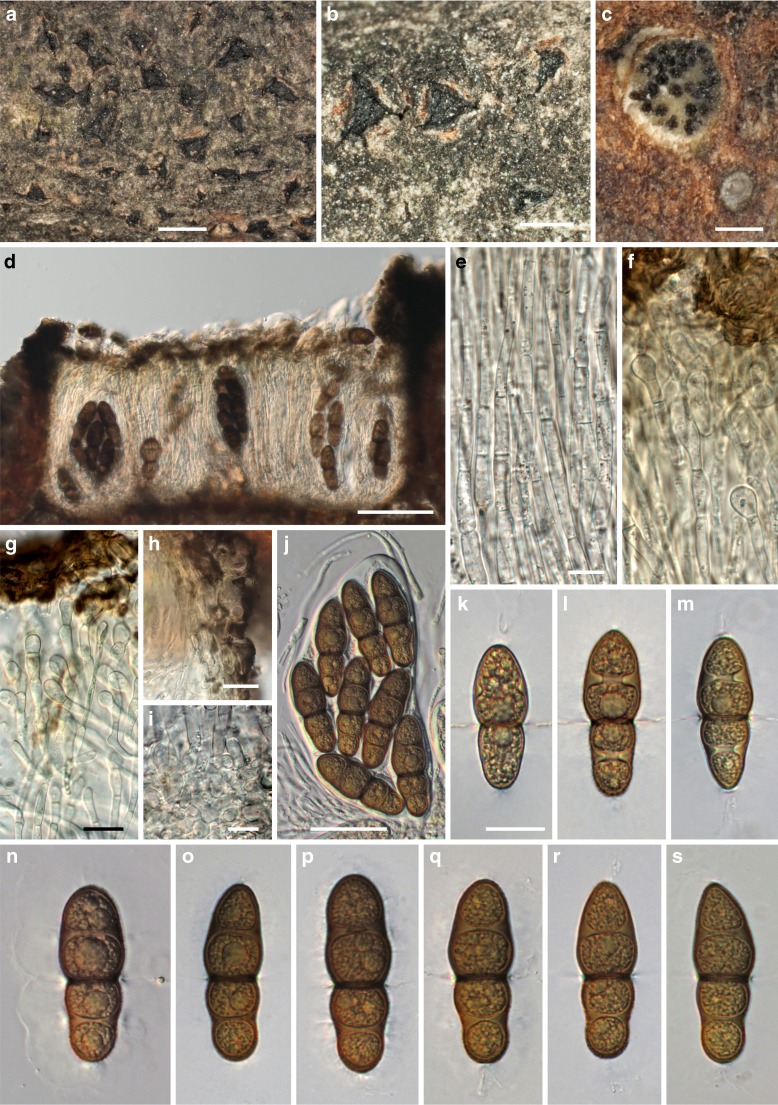
*Stigmatodiscus enigmaticus*, sexual morph. **a, b** Ascomata erumpent from bark in face view. **c** Ascoma and pycnidium (bottom right) in transverse section, showing the hymenium with asci. **d** Vertical section of apothecioid ascomata embedded in bark, showing the paraphyses and asci embedded in a gel matrix stained dark olivaceous brown below the dark brown epithecium. **e–g** Septate, apically inflated (**f, g**) paraphyses, covered by a dark brown epithecium (**f, g**). **h** Dark brown cells of apothecium margins. **i** Subhymenium and bases of paraphyses. **j** Ascus. **k–s** Ascospores; **k–m** immature showing the central euseptum and the developing additional distosepta, **n–s** mature, in **n** showing gel sheath. All in water. *Sources*: *a*, *b*, *j*, *o*–*s*. WU 35914 (holotype); *c*, *g*, *i*. WU 35925; *d*–*f*, *h*. WU 35931; *k*, *l*. WU 35917; *m*. WU 35915; *n*. WU 35913. *Scale bars*: *a* = 1 mm. *b* = 500 μm. *c* = 200 μm. *d* = 100 μm. *e*, *f*, *i* = 10 μm. *g*, *h* = 20 μm. *j* = 50 μm. *k*–*s* = 20 μm

**Fig. 6:**
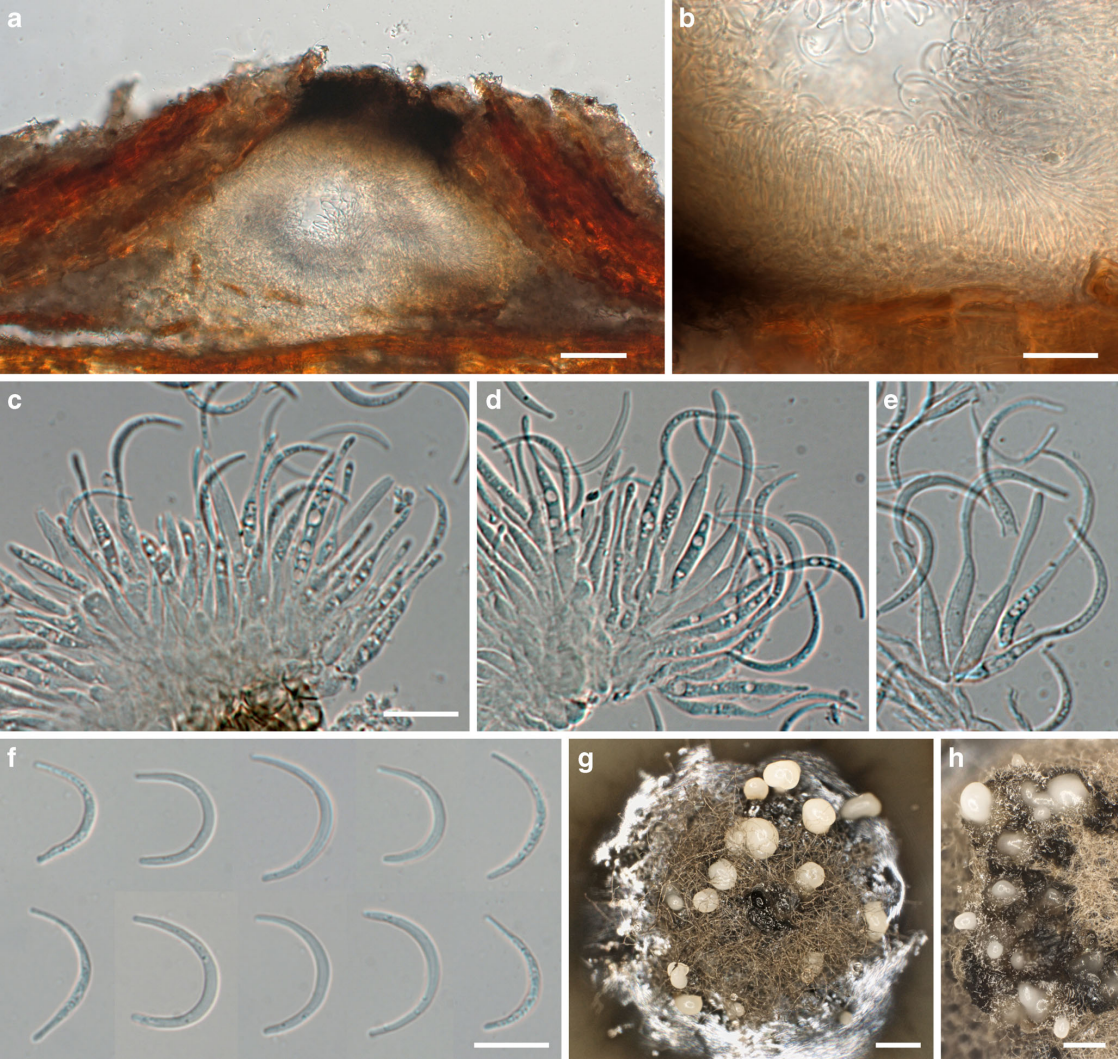
*Stigmatodiscus enigmaticus*, asexual morph. **a** Conidioma (pycnidium) immersed in periderm. **b** Detail of pycnidium with densely aggregated conidiogenous cells and conidia. **c–e** Phialides producing conidia. **f** Falcate conidia. **g, h** Acervular conidiomata on MEA showing conidial drops. All in water. *Sources*: *c*, *d*, *f*–*g*. WU 35922; *a*, *b*, *e*. WU 35925. *Scale bars*: *a* = 100 μm. *b* = 20 μm. *c*–*f* = 10 μm. *g*, *h* = 400 μm

MycoBank MB 815328, Facesoffungi number: FoF 01655, Figs. 5, 6.

*Etymology*: Referring to the uncertainties in classification when first observed.

*Ascomata* apothecioid, embedded in cortex of dead twigs, initially covered by bark, emerging through irregular cracks, eventually becoming fully exposed, dark brown to black, variable in size, 0.4–1.5 mm diam, without distinct margin. *Subhymenium* thin, ca 25–30 μm thick, of tightly packed hyaline, 3–8 μm wide hyphae. *Paraphyses* 180–300 μm long, 3–5.5 μm wide, free apical ends swollen up to 9 μm, covered by a dark brown amorphous incrustation. *Asci* (124–)152–205(−257) × (46–)54–73(−81) μm (*n* = 33), variable in shape from broadly fusoid to saccate, thick-walled, containing 8 irregularly bi- to triseriate ascospores. *Ascospores* (45.8–)54.2–64.2(−73) × (16.5–)20.0–24.3(−32.5) μm, l/w = (2.2–)2.5–2.9(−3.6) (*n* = 228), brown, asymmetric, first 1-septate, developing 2 additional distosepta and becoming 3-septate with age, constricted at the septa, surrounded by a thick gelatinous sheath, wall distinctly verruculose, dark brown, the contents granular, often with a large and several smaller guttules per cell.

*Conidiomata* on the natural substrate associated with ascomata, immersed, peridermal, pycnidial, depressed, unilocular, of circular to irregular shape, opening in irregular bark cracks, 200–500 μm diam, wall thin, inconspicuous, ca. 10–15 μm, composed of hyaline cells. *Ostiole* dark brown. *Condiogenous* cells phialidic, cylindrical, (11.2–)13.0–17.3(−21.7) × (1.5–)2.0–3.0(−3.7) μm (*n* = 40). *Conidia* falcate, (9.3–)13.0–17.0(−18.7) × (1.0–)1.3–1.7(−2.0) μm (*n* = 99), not germinating on 2 % MEA. On 2 % MEA forming superficial, aggregated, 250–400 μm wide acervuli, conidiogenous cells and conidia similar to those on the natural substrate. *Conidiophores* branched. *Conidiogenous* cells (13.5–)14.3–25.2(−30) × (1.1–)1.5–2.5 μm (*n* = 10). *Conidia* (9.0–)12.7–19.7(−24.2) × (1.0–)1.1–1.5(−1.8) (*n* = 40). *Cultures* on 2 % MEA very slow-growing, reaching 14 mm diam after one month at room temperature, dark grey to black, at age with whitish aerial hyphae in the centre, reverse first brown, then black.

*Holotype*: AUSTRIA, Wien, Landstraße, Botanical Garden of the University (HBV), on *Acer monspessulanum*, 14 Oct. 2010, H. Voglmayr (WU 35914, ex-type culture CBS 132036 = L69).

*Additional specimens examined*: AUSTRIA, Niederösterreich, Gumpoldskirchen, near Richardshof, on *Acer campestre*, 24 May 2015, H. Voglmayr & I. Greilhuber (WU 35931). Wien, Donaustadt, Lobau, Panozzalacke, on *Acer campestre*, 4 Feb. 2012, H. Voglmayr (WU 35913, culture L84). Landstraße, Botanical Garden of the University (HBV), on *Acer monspessulanum*, 7 May 2015, H. Voglmayr (WU 35925). CROATIA, Istria, Krnica, on *Carpinus orientalis*, 25 Sep. 2010, H. Voglmayr & W. Jaklitsch (WU 35915, culture L68). St. Golaš, on *Carpinus orientalis*, 31 Oct 2010, H. Voglmayr (WU 35916, culture L71 = CBS 131997). CZECH REPUBLIC, Morava, Lednice, Arboretum, on *Acer monspessulanum*, 9 Oct 2010, H. Voglmayr & W. Jaklitsch (WU 35917, culture L64). FRANCE, Provence-Alpes-Côte d’Azur, Dept. Alpes-de-Haute-Provence (04), Prieuré de Ganagobie, on *Acer monspessulanum*, 30 Jul 2011, H. Voglmayr (WU 35918, culture L76 = CBS 132037). Dept. Var (83), Gorges du Verdon, Pont de l’Artuby, on *Acer monspessulanum*, 28 Jul 2011, H. Voglmayr (WU 35919, culture L75). GREECE, Crete, Chania, Omalos, 920 m, 35.37° N, 23.897° E, on *Acer sempervirens*, 26 Nov 2011, W. Jaklitsch (WU 35911, culture L82). ibid., 5 June 2015, H. Voglmayr & W. Jaklitsch (WU 35932). Omalos, 1040 m, 35.362° N, 23.908° E, on *Acer sempervirens*, 28 Nov 2011, W. Jaklitsch (WU 35912, culture L83). ibid., 5 June 2015, H. Voglmayr & W. Jaklitsch (WU 35933). Chania, Askifou, E Karés, 790 m, 35.296° N, 24.209° E, on *Acer sempervirens*, 6 June 2015, H. Voglmayr & W. Jaklitsch (WU 35934). ENE Karés, 670 m, 35.305° N, 24.204° E, on *Acer sempervirens*, 6 June 2015, H. Voglmayr & W. Jaklitsch (WU 35935). Rhetimno, Psiloritis, S Anogia, 930 m, 35.274° N, 24.886° E, on *Acer sempervirens*, 8 June 2015, H. Voglmayr & W. Jaklitsch (WU 35936). Psiloritis, S Anogia, 1490 m, 35.22° N, 24.87° E, on *Acer sempervirens*, 8 June 2015, H. Voglmayr & W. Jaklitsch (WU 35937). ITALY, Lazio, Viterbo, Norchia, on *Acer campestre*, 14 Oct 2013, H. Voglmayr, W. Jaklitsch & W. Gams (WU 35920, culture L122). Montalto di Castro, Vulci, on *Acer monspessulanum*, 15 Oct 2013, H. Voglmayr, W. Jaklitsch & W. Gams (WU 35923). SLOVENIA, Sežana, Skocjan, on *Acer monspessulanum*, 17 May 2015, H. Voglmayr & I. Greilhuber (WU 35922, culture L162).

Notes: The sequences of the collections from *Acer sempervirens* in Crete deviate slightly from the other collections; however, morphological features match the species. The variability is therefore considered to be within the intraspecific range of the species and may be the result of genetic isolation on a Mediterranean island.

## Discussion

Phylogenetic analyses place *Stigmatodiscales* within *Dothideomycetes* (Fig. 1), but their closest relatives remain obscure and unresolved. A weakly support relationship to *Monoblastiales*, *Dyfrolomycetales* and *Acrospermales* is only revealed in ML analyses, and these orders are morphologically and ecologically highly distinct (Hyde et al. 2013; Pang et al. 2013).

Morphologically, the two new genera share a unique combination of characters within *Dothideomycetes*: they have apothecial ascomata embedded in host tissue lacking an excipulum, saccate fissitunicate asci, large, 3-septate ascospores with an excentric euseptum and two additional distosepta, being surrounded by a large gelatinous sheath. Ascospores of *Stigmatodiscus* resemble those of *Stigmatomassaria* and are to our knowledge unknown in other groups of *Dothideomycetes*. Some species of *Lichenothelia* have brown asymmetric ascospores with one euseptum and two distosepta surrounded by a large gelatinous sheath (Hawksworth 1981). However, the ascomata of *Lichenothelia* are quite unlike those of *Stigmatodiscales*; they are superficial and perithecioid when young, becoming apothecioid (lecideine) at maturity with an excipulum composed of pseudoparenchymatous tissue (Hawksworth 1981; Øvstedal and Lewis Smith 2001). In addition, in *Lichenothelia* species with 3-septate ascospores these are much smaller (less than 30 μm long) and commonly become submuriform by additional transverse septa (Hawksworth 1981; Henssen 1987). Ecologically, *Lichenothelia* differs by inhabiting rocks, being often found in association with algae or with lichen thalli (Øvstedal and Lewis Smith 2001; Muggia et al. 2012). Ascospores similar to those of *Asterodiscus* can be also found in other groups, particularly in the *Arthopyreniaceae* of the *Pleosporales*, but ascomata of members of this family do not open to become apothecial (Hyde et al. 2013). Within *Trypetheliaceae*, *Architrypethelium* and *Ornatopyrenis* have similar ascospores of the same size as observed in *Stigmatodiscales*, but they are lichenized and pyrenocarpous (Aptroot 1991). In *Xylopezia* (Sherwood-Pike and Boise 1986), where ascomata are first immersed and closed, asci are functionally unitunicate and the ascospores are euseptate. The only order of the *Dothideomycetes* comprising exclusively discomycetous fungi is the *Patellariales*. However, all representatives of this order form black, drought-tolerant, more or less superficial apothecia, and their ascospores are euseptate, except in *Lirellodisca* (Kutorga and Hawksworth 1997; Yacharoen et al. 2015). All lineages discussed above for which sequence data are available are phylogenetically distant from *Stigmatodiscales* (Fig. 1).

Although ascospores of the *Stigmatodiscales* are highly similar to those of the genera *Asteromassaria* and *Stigmatomassaria*, the fungi of the present study are phylogenetically remote from these. *Asteromassaria* and *Stigmatomassaria* are both contained within *Pleomassariaceae*, *Pleosporales* (unpubl. data), whereas the fungi of the present study form a highly supported clade within *Dothideomycetes*, which is of uncertain phylogenetic affinity (Fig. 1). In addition, ascomata of *Asteromassaria* and *Stigmatomassaria* are perithecioid pseudothecia typical of *Pleomassariaceae* (Barr 1982). Acknowledging their profound phylogenetic and morphological distinctness, classification within two new genera in a new family and order is fully justified.

The genera *Asterodiscus* and *Stigmatodiscus* are similar in their ascomatal traits. The main differences concern the ascospore shape and colouration, analogous to the closely related genera *Asteromassaria* and *Stigmatomassaria*. *Asterodiscus* has hyaline, only slightly asymmetric ascospores which become brownish only after ejection, whereas *Stigmatodiscus* has dark brown, asymmetric ascospores with the distal part distinctly larger than the proximal part.

Ecologically, species of *Asterodiscus* and *Stigmatodiscus* are characterised by growth on recently dead corticated twigs still attached to the trees. While *Asterodiscus* appears to be confined to *Tamarix* spp., *Stigmatodiscus* has been primarily observed on various *Acer* species (*A. campestre*, *A. monspessulanum*, *A. sempervirens*) but also on the unrelated *Carpinus orientalis*. *Asterodiscus tamaricis* and *Stigmatodiscus enigmaticus* are widely distributed in Central and Southern Europe and have been commonly observed during the current study on various *Tamarix* spp. and *Acer monspessulanum*, respectively. Occurrence on exposed twigs primarily in submediterranean climate indicates a high level of drought tolerance. It is remarkable that they have remained undetected up to date on these widely distributed hosts, demonstrating once again the need of detailed biodiversity studies on corticolous ascomycetes especially in Central and Southern Europe (e.g. Voglmayr and Jaklitsch 2008, 2011, 2014; Jaklitsch and Voglmayr 2011, 2014; Voglmayr et al. 2012; Jaklitsch et al. 2013, 2014, 2015; Galán et al. 2015; Checa et al. 2015).
